# Epidermal second-hit mutation in *MVK* gene associated with linear porokeratosis

**DOI:** 10.1016/j.gendis.2024.101314

**Published:** 2024-04-29

**Authors:** Anqi Zhao, Xinyi Wang, Chaolan Pan, Yumeng Wang, Qiaoyu Cao, Min Li, Ming Li

**Affiliations:** Department of Dermatology, The Children's Hospital of Fudan University, Shanghai 201102, China; Department of Dermatology, Xinhua Hospital, Shanghai Jiaotong University School of Medicine, Shanghai 200092, China; Institute of Dermatology, Shanghai Jiaotong University School of Medicine, Shanghai 200092, China; Department of Dermatology, Minhang Hospital, Fudan University, Shanghai 201199, China; Department of Dermatology, Xinhua Hospital, Shanghai Jiaotong University School of Medicine, Shanghai 200092, China; Institute of Dermatology, Shanghai Jiaotong University School of Medicine, Shanghai 200092, China; Department of Dermatology, The Children's Hospital of Fudan University, Shanghai 201102, China; Department of Dermatology, Dushu Lake Hospital Affiliated to Soochow University (Medical Center of Soochow University, Suzhou Dushu Lake Hospital), Suzhou, Jiangsu 215000, China; Department of Dermatology, The Children's Hospital of Fudan University, Shanghai 201102, China

Porokeratosis encompasses a group of keratinization disorders with distinct clinical variants, including porokeratosis of Mibelli, disseminated superficial actinic porokeratosis, porokeratosis plantaris, palmaris et disseminata, and linear porokeratosis (LP).^1^ Familial porokeratosis has been associated with pathogenic variants in genes of the mevalonate pathway (such as *MVK*, *PMVK*, *MVD*, and *FDPS*), a vital metabolic pathway responsible for synthesizing sterols and isoprenoid metabolites.^1^, ^2^, ^3^

LP is characterized by limited, segmental keratinized skin lesions along with Blaschko's line, typically appearing in infancy or early childhood. Recent studies by Atzmony et al and Kubo et al identified second-hit postzygotic mutation events of *PMVK* and *MVD* in LP patients, supporting the hypothesis of second-hit mutation in LP.^4^^,^^5^ Pathogenic mutations involve a heterozygous germline mutation and a second-hit event in affected skin, including loss of the wild-type allele (loss of heterozygosity, LOH) or compound heterozygosity. In our study, we reported a case of 6-year-old boy diagnosed with LP and identified a novel germline heterozygous *MVK* c.389_394del: p.D130_I131del mutation, along with somatic LOH confined to the lesional epidermis. This study represents the first report linking *MVK* mutation to LP pathogenesis.

Our patient presented with linear brown plaques on his right underarms, hips, and lower limbs, with mildly pruritic. The plaques initially appeared as small patches when he was 2 years old, progressively enlarged and fused to form linear lesions over time (Fig. 1A). His family members didn't have similar syndromes. Histopathological examination revealed the characteristic cornoid lamellae in the stratum corneum and the absence of granular layer beneath it, melanophages and perivascular lymphocytes infiltration in the upper dermis (Fig. 1C). Immunofluorescence staining revealed an upregulation of the epidermal basal layer marker keratin 14 and terminal differentiation marker transglutaminase 1 in patient, with abnormal distribution of terminal differentiation marker transglutaminase 1 in the cornoid lamellae, suggesting enhanced epidermal proliferation and abnormal differentiation (Fig. S1).

**Figure 1:**
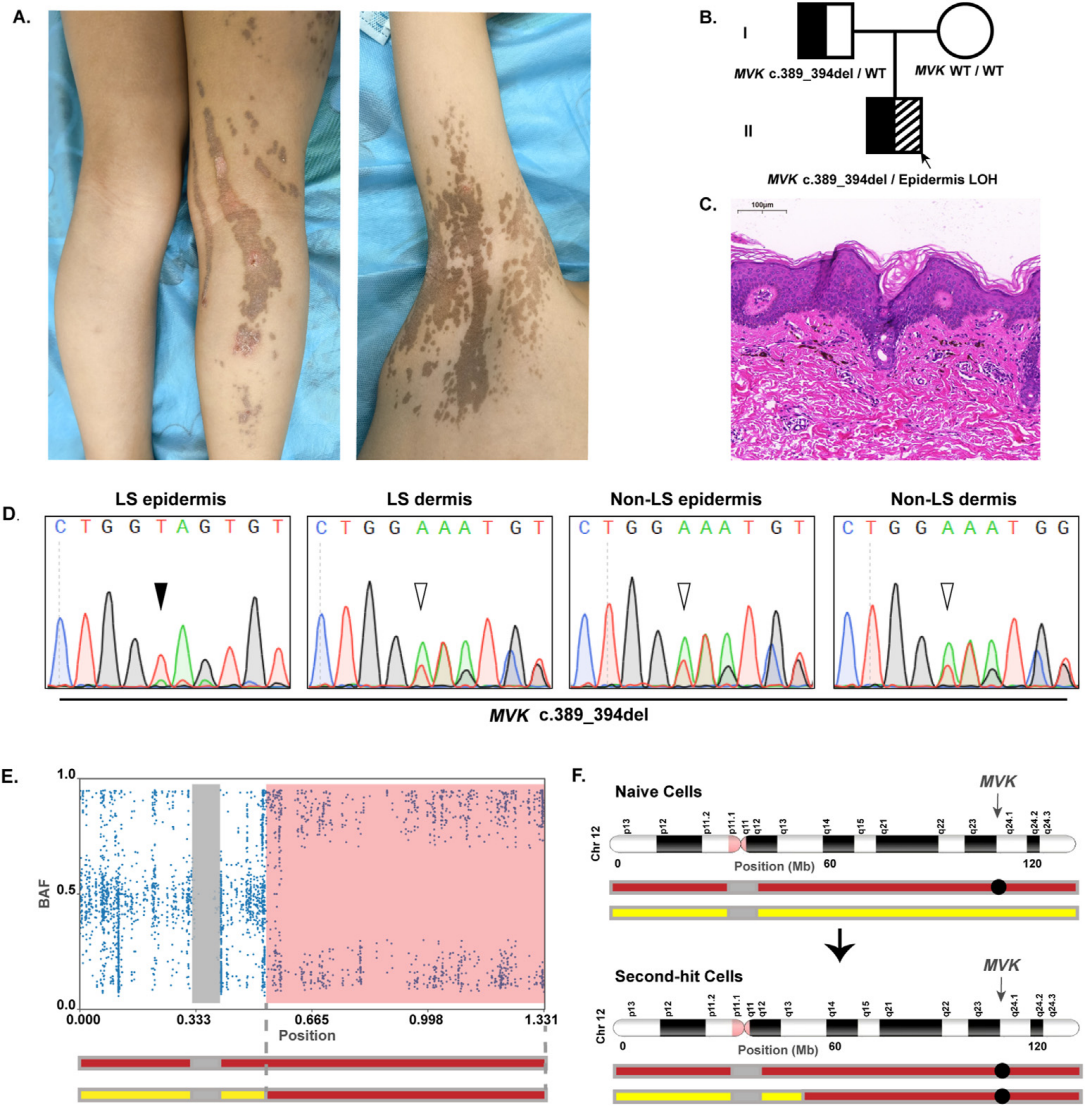
Epidermal second-hit mutation in *MVK* detected in a patient with linear porokeratosis. **(A)** Skin manifestations of patient on the right underarms and lower limbs. **(B)** The family genogram, the arrow indicated the proband. **(C)** Hematoxylin and eosin staining of the lesional skin of patient showing cornoid lamella in the stratum corneum, melanophages, and perivascular lymphocytes infiltration in the upper dermis. Scale bars, 100 μm. **(D)** Sanger sequencing revealed the heterozygous c.389_394del mutation of *MVK* (white arrowhead) and LOH (black arrowheads) in the lesional epidermis samples of patient. **(E)** BAF of Chr12 in the lesional epidermis of lesional skin sample. The LOH region is shown in pink in the BAF plot. Gray bar indicates the uncovered region of the centromere. (**F)** Schematic of the second-hit mutation on Chr 12 in lesional epidermis. Chr12 containing the congenital *MVK* mutation (black circle) and wild-type *MVK* are indicated by the red and yellow bars, respectively. Postzygotic LOH in chr12q rendered the monoallelic c.389_394del mutation of *MVK* biallelic. BAF, B-allele frequency; Chr, chromosome; LS, lesional skin; non-LS, non-lesional skin; LOH, loss of heterozygosity.

To identify the pathogenic mutation, we initially conducted panel sequencing targeting ∼500 causative genes of genodermatoses on DNA, which were obtained from full-thickness lesional skin biopsy. A mosaic *MVK* c.389_394del mutation with a ratio of 59.23% was identified (wild type to mutant type ratio of 243:353, Table S1). To pinpoint the exact pathogenic mutation, we divided the lesional skin biopsy into epidermis and dermis using Dispase II treatment and performed genodermatoses panel sequencing again on DNA obtained from both the epidermis and dermis (Table S2). Sanger sequencing was performed to validate the results. We identified a heterozygous *MVK* (NM_000431.4) c.389_394del mutation in lesional dermis and non-lesional epidermis and dermis. Interestingly, a higher mutant allele fraction in the lesional epidermis was detected, suggesting somatic LOH (Fig. 1D). Whole-exome sequencing of DNA obtained from lesional epidermis further confirmed the epidermis-specific LOH at the terminal ∼70 Mb region of chromosome 12q, which included the *MVK* gene locus (Fig. 1E). In addition, Sanger sequencing was performed on the blood samples of the proband and his parents, showing a validate heterozygous germline mutation in the patient and his father (Fig. S2). Family genogram was shown in Figure 1B. These results indicate that LOH in chromosome 12q rendered the monoallelic c.389_394del mutation of *MVK* biallelic (Fig. 1F). Detailed materials and methods were described in supplementary materials.

We report a novel *MVK* mutation c.389_394del: p.D130_I131del, involving the deletion of bases 389 to 394, which resulted in the absence of aspartic acid and isoleucine encoded by codons 130 and 131, respectively. Residue 132 is known as an ATP-binding site in the MVK protein. Therefore, we hypothesize that the loss of amino acids at positions 130 and 131 may affect MVK's interaction with ATP, and consequently impact the function of the MVK protein. As previously reported, linear porokeratosis requires biallelic loss-of-function mutations. The heterozygous germline mutation *MVK* c.389_394del inherited from the subject's father does not cause skin disease since a wild type copy of *MVK* is also present. Only in the stripe of affected skin, where second-hit LOH makes this presumed biallelic loss-of-function mutation, does disease ensue. To date, no reports have documented the incomplete external manifestation in linear porokeratosis. This observation underscores the need for further research to explore the prevalence and significance of this phenomenon.

In conclusion, our research represents the first report linking postzygotic second-hit *MVK* mutation to LP pathogenesis, significantly expanding the genotype spectrum of LP. Importantly, we emphasize the importance of isolating the epidermis to detect postzygotic mutations during epidermis development. Additionally, it is conceivable that the second-hit events within the dermis occur at frequencies beneath the detection threshold of current sequencing technologies. Understanding this process is crucial for gaining deeper insights into the pathogenesis of linear porokeratosis and may pave the way for targeted therapeutic interventions in the future.

## Ethics declaration

This study was approved by the ethics committees of Children's Hospital of Fudan University (No. (2023) 31). Written informed consent for publication of their clinical details and clinical images was obtained from the parents of patient.

## Author contributions

Ming Li designed the study and approved the final version. Anqi Zhao and Xinyi Wang contributed to the collection of participants' samples and clinical data. Anqi Zhao drafted the manuscript; Xinyi Wang and Min Li revised the manuscript; Anqi Zhao, Chaolan Pan, Yumeng Wang, and Qiaoyu Cao participated in the experiments. All authors participated in the discussion of the results. The authors read and approved the final manuscript.

## Data availability

All data described in this manuscript are available.

## Conflict of interests

The authors declared that they have no conflict of interests.

## Funding

This work was supported by grants from the National Natural Science Foundation of China (No. 82073422, 82273504).
