# Supplementary material for: Epidermal second-hit mutation in MVK gene associated with linear porokeratosis
Source: Genes Dis. 2024 Apr 29;12(1):101314. doi: 10.1016/j.gendis.2024.101314 (PMC11462185; doi:10.1016/j.gendis.2024.101314)
Supplement: Multimedia component 1 [file mmc1.docx]

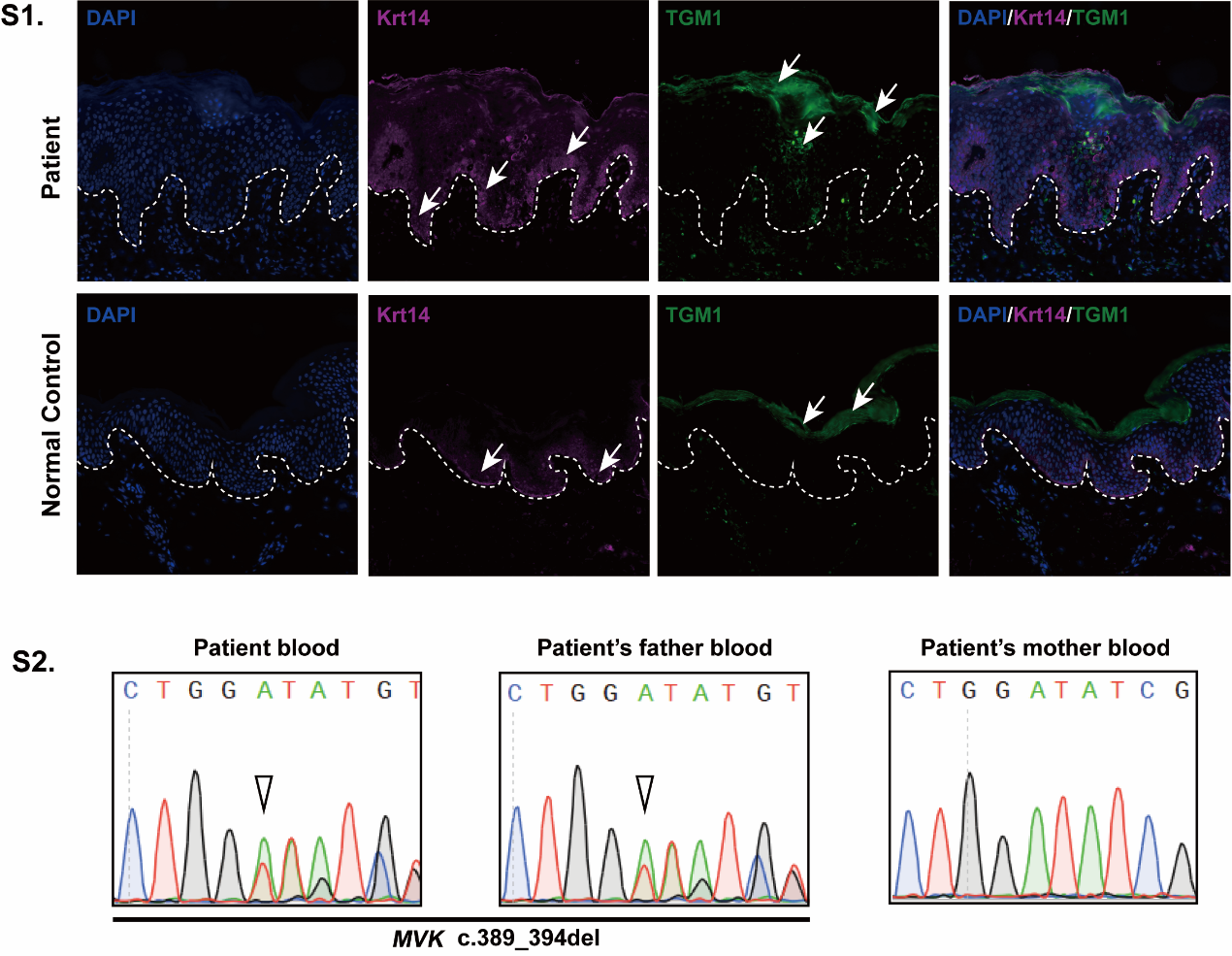
**Figure S1. Abnormal proliferation and differentiation in lesional skin. Figure S1. Fig S1. Abnormal proliferation and differentiation in lesional skin.** Immunofluorescence staining revealed the upregulation of the epidermal basal layer marker keratin 14 (Krt14) and terminal differentiation marker transglutaminase 1 (TGM1) in patient. Scale bars, 185mm.

**Figure S2. Mutation analysis of the family.**


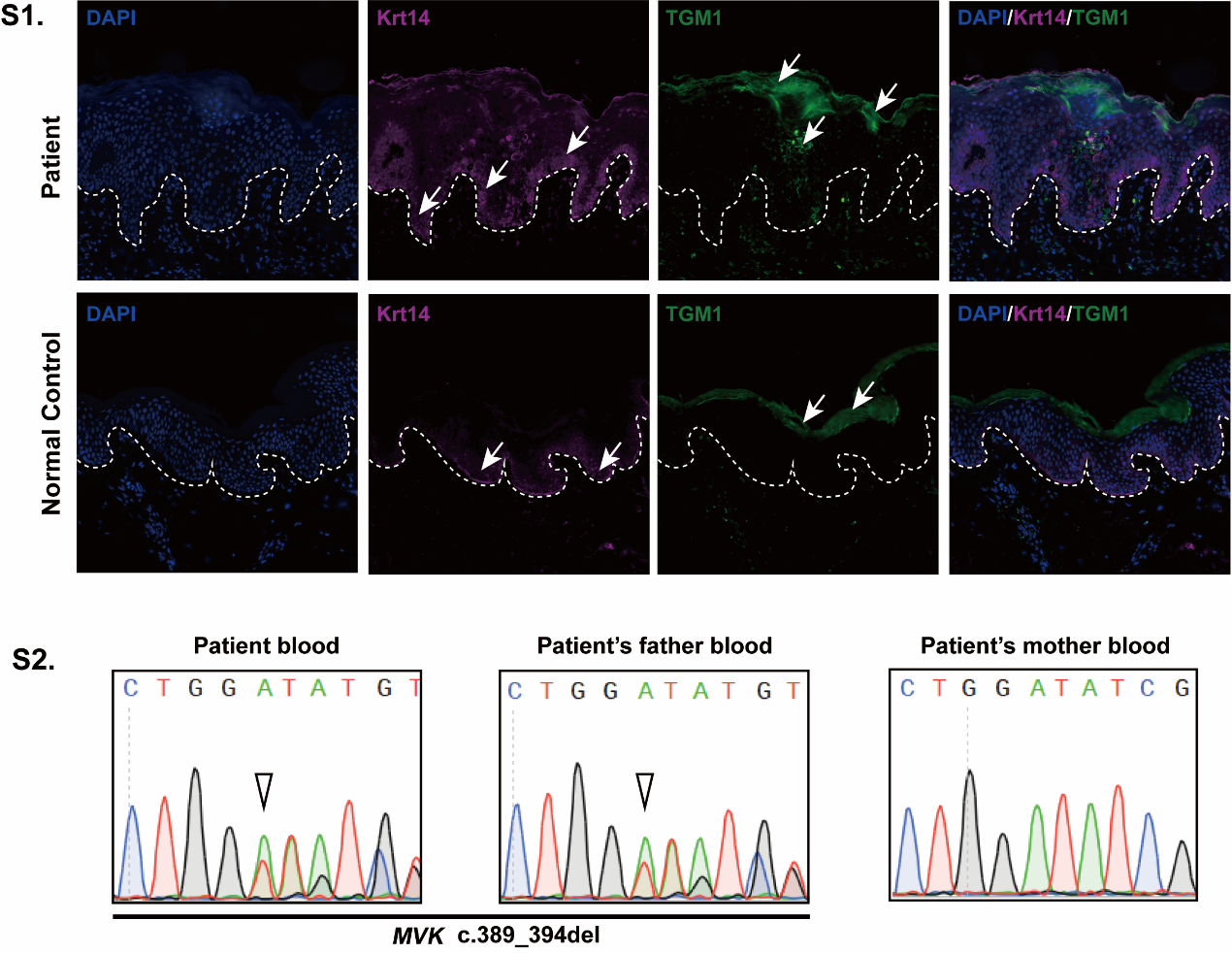


**Fig S2. Mutation analysis of the family.** Sanger sequencing revealed the heterozygous c.389_394del mutation of *MVK* in patients’ and his father’s blood.

**Detailed materials & methods：**

1. **Sample collection**

This study was approved by the ethics committees of Children’s Hospital of Fudan University [No. (2023) 31]. Written informed consent for publication of their clinical details and clinical images was obtained from the parents of patient. Serum samples were collected from proband and his parents. Skin punch biopsies (0.5*0.5*0.6 mm) were collected from lower leg skin of the proband, containing lesional and non-lesional skin. Normal skin samples were materials obtained during surgery to remove pigmented nevus of subjects.

1. **Dispase II treatment, DNA isolation, and genetic analysis**

Lesional skin punch biopsies were bisected, with one portion designated for full-thickness skin DNA extraction, and the other subjected to enzymatic dissociation using 2% Dispase II (Merck, Cat No. 04942078001) at 4°C overnight to facilitate separation into epidermal and dermal layers. Similarly, non-lesional skin punch biopsies underwent enzymatic treatment with 2% Dispase II at 4°C overnight, leading to their division into epidermis and dermis components.

DNA was isolated from the blood sample and skin biopsy according to the manufacturer’s instructions. Panel sequencing targeting ~500 causative genes of genodermatoses was performed to detect potential pathogenic mutations (The included causative genes were listed in **Table S3**). The exons and their adjacent splicing regions (approximately 20 bp) of these targeted genes were captured and enriched for sequencing on an Illumina HiSeq 2000 platform, achieving a depth of coverage of 500x. Disease-causing variants were identified by comparing with the reported cDNA reference sequence (GenBank accession no. NM_001651.3). Sequence variants were filtered against the public databases (gnomAD, OMIM, HGMD, and ClinVar). SIFT and Polyphen2 were used to predict the functional effects of expressed variants. Confirmation of significant findings was achieved through Sanger sequencing.

For the detection of chromosomal segments exhibiting loss of heterozygosity (LOH), whole-exome sequencing (WES) was conducted on DNA isolated from lesional epidermal biopsies. LOH analysis involved the computation of B-allele frequencies, which entailed dividing the count of non-reference (B-allele) reads by the total number of reads for each single nucleotide variant (SNV) identified. This calculation was performed using custom scripts in Python and R. The B-allele frequency data were then graphically represented, plotting these frequencies against their corresponding genomic locations, to visually depict regions of LOH across the chromosomes.

1. **Immunofluorescence staining**

Immunofluorescences staining experiments were conducted following the manufacturer's instructions (Cell Signaling Technology, USA). Paraffin-embedded skin sections were hydrated sequentially in xylene and gradient ethanol. Sections were blocked with 10% horse serum in PBS at room temperature for 1 h using 10% horse serum in PBS. Primary antibody was added to the sections and incubated overnight at 4°C. After that, secondary antibodies were added and incubated at room temperature for 1 hour. The antibodies and their dilutions used in this study were TGM1 antibody (1:100, Proteintech, Cat No. 12912-3-AP), Krt14 Rabbit mAb (1:200, Abclonal, Cat No. A19039).
